# Exercise-Induced Circulating microRNAs: Potential Key Factors in the Control of Breast Cancer

**DOI:** 10.3389/fphys.2022.800094

**Published:** 2022-06-16

**Authors:** Guilherme Defante Telles, Miguel Soares Conceição, Felipe Cassaro Vechin, Cleiton Augusto Libardi, Marcelo Alves da Silva Mori, Sophie Derchain, Carlos Ugrinowitsch

**Affiliations:** ^1^ Laboratory of Neuromuscular Adaptations to Strength Training, School of Physical Education and Sport, University of São Paulo (USP), São Paulo, Brazil; ^2^ Department of Obstetrics and Gynecology, Faculty of Medical Sciences, University of Campinas, Campinas, Brazil; ^3^ MUSCULAB—Laboratory of Neuromuscular Adaptations to Resistance Training, Department of Physical Education, Federal University of São Carlos (UFSCar), São Carlos, Brazil; ^4^ Department of Biochemistry and Tissue Biology, Institute of Biology, University of Campinas, Campinas, Brazil; ^5^ Obesity and Comorbidities Research Center (OCRC), University of Campinas, Campinas, Brazil; ^6^ Experimental Medicine Research Cluster (EMRC), Campinas, Brazil

**Keywords:** skeletal muscle, breast cancer, disease, epigenetics, miRNA, tumor, crosstalk, intercellular communication

## Abstract

Losses in skeletal muscle mass, strength, and metabolic function are harmful in the pathophysiology of serious diseases, including breast cancer. Physical exercise training is an effective non-pharmacological strategy to improve health and quality of life in patients with breast cancer, mainly through positive effects on skeletal muscle mass, strength, and metabolic function. Emerging evidence has also highlighted the potential of exercise-induced crosstalk between skeletal muscle and cancer cells as one of the mechanisms controlling breast cancer progression. This intercellular communication seems to be mediated by a group of skeletal muscle molecules released in the bloodstream known as myokines. Among the myokines, exercise-induced circulating microRNAs (c-miRNAs) are deemed to mediate the antitumoral effects produced by exercise training through the control of key cellular processes, such as proliferation, metabolism, and signal transduction. However, there are still many open questions regarding the molecular basis of the exercise-induced effects on c-miRNA on human breast cancer cells. Here, we present evidence regarding the effect of exercise training on c-miRNA expression in breast cancer, along with the current gaps in the literature and future perspectives.

## Introduction

Significant loss of skeletal muscle mass, strength, and metabolic function is associated with unfavorable prognosis in various cancer types, including breast cancer (Rier et al., 2016; Kubo et al., 2017; Caan et al., 2018; Jang et al., 2020; Lee et al., 2021). Decreases in skeletal muscle strength and mass impair overall health of patients with breast cancer due to enhanced drug toxicity and cancer-related fatigue (Kazemi-Bajestani et al., 2016; Murphy, 2016; Boekel et al., 2018; Guigni et al., 2018; Mazzuca et al., 2018), which require easing the treatment regimen. Together, these factors can increase morbidity and mortality of patients with cancer (Kazemi-Bajestani et al., 2016; Zhang et al., 2020). Contrarily, physical exercise training is one of the most effective strategies to increase skeletal muscle strength and mass, metabolic function, overall health, and quality of life of patients with breast cancer (Garber et al., 2011; Hawley et al., 2014; Dethlefsen et al., 2017a; Adraskela et al., 2017; Kraschnewski and Schmitz, 2017; Hojman et al., 2018; Campbell et al., 2019; Hayes et al., 2019; Patel et al., 2019). Interestingly, a growing body of evidence has recently indicated that exercise training also plays a role in controlling breast cancer progression through skeletal muscle release of humoral factors in the blood stream (Hojman et al., 2011; Dethlefsen et al., 2016; Ruiz-Casado et al., 2017; Figueira et al., 2018; Hojman et al., 2018).

The exercise-induced humoral factors allow the crosstalk between skeletal muscle cells and other tissues’ cells (Pedersen and Febbraio, 2012; Hawley et al., 2014; Safdar et al., 2016; Safdar and Tarnopolsky, 2018). Those factors are known as myokines, a group of molecules (e.g, protein/peptides, metabolites and different species of RNAs) that regulates key cellular processes, such as cell proliferation, metabolism, and signal transduction, acting as important mediators of the systemic effects of exercise training (Safdar et al., 2009; Pedersen and Febbraio, 2012; Hawley et al., 2014; Dethlefsen et al., 2016; Safdar et al., 2016; Dethlefsen et al., 2017a; Dethlefsen et al., 2017b; Ruiz-Casado et al., 2017; Safdar and Tarnopolsky, 2018). Among the myokines, the microRNAs (miRNAs) have been deemed as important molecules as they regulate cellular activity at the post-transcriptional level through mRNA degradation, destabilization, or repression of gene translation (Safdar et al., 2016; Ha and Kim, 2014; Huntzinger and Izaurralde, 2011; Alizadeh et al., 2019; Camera et al., 2016; D'Souza et al., 2017; Davidsen et al., 2011; Drummond et al., 2008; Fyfe et al., 2016; Mooren et al., 2014; Nielsen et al., 2010; Ogasawara et al., 2016; Telles et al., 2021). In fact, miRNAs are a class of small (∼22 nucleotides) non-coding RNAs that can be produced in different tissues (e.g., skeletal muscle, adipose tissue) and released to the bloodstream (i.e., circulating miRNAs: c-miRNAs) (Safdar et al., 2016; Safdar and Tarnopolsky, 2018; Alizadeh et al., 2019; D'Souza et al., 2017; Davidsen et al., 2011; Drummond et al., 2008; Fyfe et al., 2016; Nielsen et al., 2010; Ogasawara et al., 2016) usually associated with different proteins and lipoprotein complexes (e.g., protein argonaute-2, high- and low-density lipoproteins or inserted in extracellular vesicles) (Safdar et al., 2016; Dufresne et al., 2018; Safdar and Tarnopolsky, 2018). As c-miRNAs are demonstrated to modulate the expression of genes related to tumor development/progression, these myokines have been proposed as key factors involved in the effects produced by exercise training on breast cancer cells (Zhang et al., 2007; Isanejad et al., 2016; Santos et al., 2016; Adhami et al., 2018; Dufresne et al., 2018; Santos et al., 2018). In the present perspective, we present evidence regarding the effect of exercise training on c-miRNA expression in breast cancer, along with current gaps in the literature and experimental design suggestions to address these gaps.

## Has the Effect of Exercise Training *per se* on c-miRNA Expression Been Investigated in Breast Cancer?

Mounting evidence indicates that exercise training can mitigate the development of breast tumors (Hojman et al., 2011; Dethlefsen et al., 2016; Figueira et al., 2018; Hojman et al., 2018). Figueira, Cortinhas (Figueira et al., 2018), in a meta-analysis of 28 preclinical studies [including 2085 animals (rats, mouse, and mice) with breast cancer], showed that exercise training promotes a reduction in the total number of tumors (-20.2%), and tumors per animal (-63.2%), as well as in tumor proliferation (-79.4%), weight (-36.6%), and volume (-44.3%). In an *in vitro* model, Hojman, Dethlefsen (Hojman et al., 2011) incubated MCF-7 breast cancer cells with the blood serum of mice following exercise and observed a 52% decrease in tumor cell proliferation. The results of an elegant study by Dethlefsen, Lillelund (Dethlefsen et al., 2016) corroborate the previous ones showing a decreased viability (∼-9.5%) of different breast cancer cells lineages (MCF-7 and MDA-MB-231) incubated with the serum of breast cancer survivors (undergoing chemotherapy) following an exercise bout when compared to resting serum. Collectively, the aforementioned findings indicate the existence of exercise-induced circulating factors with antitumoral effects. Considering their role in intercellular communication, c-miRNAs are proposed to participate in these effects (Safdar et al., 2016; Dufresne et al., 2018; Safdar and Tarnopolsky, 2018).

Isanejad, Alizadeh (Isanejad et al., 2016), using the inbred female BALB/c mice (6–8 weeks old) model of breast cancer, demonstrated that neoadjuvant hormone therapy combined with a 5-week exercise training protocol increased the expression of miRNA-206 and let-7a (both related to tumor suppression) and reduced the expression of the oncomiR miR-21 in tumor tissue. These results were accompanied by decreased ERα and HIF-1 mRNA levels (linked to tumor growth and angiogenesis) (Cheng et al., 2005; Akao et al., 2006; Liu et al., 2011), along with a reduction in Ki67 expression, an important nuclear marker of cell proliferation related to poorer survival in women with breast cancer (Cheang et al., 2009). However, the literature investigating the role of exercise-induced c-miRNAs in humans with breast cancer is scarce (Adams et al., 2018; Hagstrom and Denham, 2018; Alizadeh et al., 2019). Adams, Arem (Adams et al., 2018) compared c-miRNAs changes between patients assigned to either a weight loss intervention (encompassing increased physical activity, reduced caloric intake, and behavioral therapy) or usual care. Significant differences in the expression of six c-miRNAs were observed in the weight loss arm: an increase in miR-191-5p, -24-3p and let-7b-5p, and a decrease in miR-106b-5p, -27a-3p, and -92a-3p. Interestingly, the usual care arm showed changes in the opposite direction for the expression of miR-106b-5p, -191-5p, and 92a-3p. Most notably, miR-106 family supports tumor cells proliferation (Ivanovska et al., 2008) and is used for prognosis of cancer recurrence (Zheng et al., 2015). Importantly, patients underwent a weight loss intervention encompassing increased physical activity, reduced caloric intake, and behavioral therapy, and patients had completed their treatment before enrolment. Hagstrom and Denham (Hagstrom and Denham, 2018) showed that 16 weeks of resistance exercise training did not produce significant changes in c-miRNA expression compared to usual care in patients with stage I–IIIA breast cancer participants who completed the treatment and had no evidence of recurrent disease (except for hormonal therapy). However, high-responders (based on the gains in upper and lower body strength) to the resistance exercise training exhibited increased c-miR-133a-3p relative to low responders. The miR-133a may have an important tumor suppression effect regulating cell cycle and breast cancer cell proliferation (Cui et al., 2013). Alizadeh, Isanejad (Alizadeh et al., 2019) investigated the response of exercise training on c-miRNA levels in women with hormone receptor-positive breast cancer treated with hormone therapy. Patients were divided into two groups: hormone therapy and hormone therapy-exercise training. Additionally, two groups of healthy women were included in the study (negative control and positive control that performed the same exercise training protocol). Both breast cancer groups had higher baseline levels of circulating oncomiRs (miR-21, miR-155, miR-221, miR-27a, and miR-10b), as well as lower levels of tumor suppressors c-miRNAs (miR-206, miR-145, miR-143, miR-9, and let-7a) when compared to the healthy groups. Importantly, the hormonal therapy-exercise training group showed lower expression of all the analyzed c-oncomiRs compared to pre-intervention and to the hormonal therapy group (except for miR-221) at post-intervention. In addition, the authors showed a greater increase in tumor suppressor c-miRNAs related to the former than the latter (reaching values close to those of healthy women). It is noteworthy that Alizadeh, Isanejad (Alizadeh et al., 2019) study combined the exercise training intervention with hormone therapy. Taken together, no experimental design has attempted to determine the effect of exercise training *per se* on c-miRNAs expression in patients with breast cancer.

## Acute vs. Chronic Effect of Exercise Training on c-miRNA Expression in Breast Cancer

Initially, exercise training studies have focused on the chronic effect (i.e., overall training intervention effect with no residual effect from an exercise bout) of exercise interventions (i.e., exercise training) on reducing breast cancer risk factors (e.g., sex steroid hormones, inflammatory cytokines, adipokines) (McTiernan, 2008; Hojman et al., 2017). However, exercise training-induced physiological adaptations are caused by the accumulation of transient and coordinated transcriptional, translational and post-translational changes following each exercise bout (i.e., acute effect) (Hawley et al., 2014). Thus, this acute effect has been deemed as one of the main mechanisms underpinning the exercise effect on breast cancer cells.

The previously cited reduction in tumor cell proliferation occurred when MCF-7 breast cancer cells were incubated with blood serum collected immediately after mice completed an exercise bout (Hojman et al., 2011). Similarly, Dethlefsen, Lillelund (Dethlefsen et al., 2016) observed a significant reduction in the viability of MCF-7 and MDA-MB-231 breast cancer cell lines incubated with blood serum collected immediately after an exercise bout compared to the pre-exercise blood samples. Importantly, breast cancer survivors (stages I to III) resting serum, collected after 6 months of an exercise training intervention (carried out after chemotherapy completion), did not induce changes in the viability of both cell lineages. Based on these findings, the authors proposed a model in which the exercise-induced antitumoral effect is driven by the repetitive and transient acute spikes in the synthesis of systemic factors following each exercise bout (Dethlefsen et al., 2017a; Hojman et al., 2018). In this model, the response to each exercise bout (e.g., the release of systemic factors, sympathetic activation, increased blood flow) can induce immediate stress on tumor cell homeostasis, which might lead to tumoral modifications (e.g., improved immunogenicity and metabolism adjustments) and contribute to slow down tumor progression over time (Hojman et al., 2017). Although preclinical data suggest that acute exercise-induced systemic changes can control breast cancer cell development, the molecular candidates remain to be better characterized.

**FIGURE 1:**
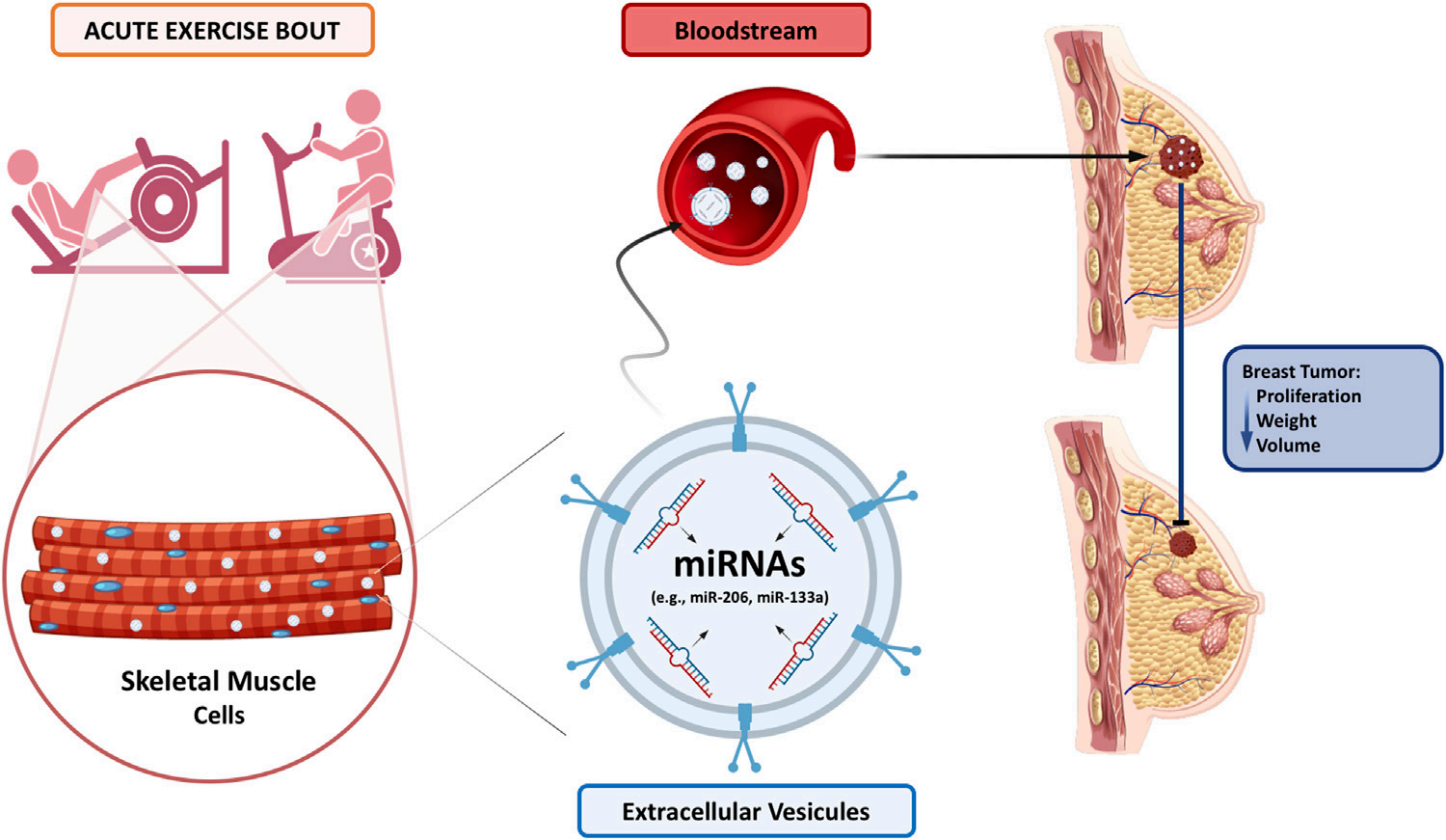
Hypothesized mechanism of exercise training controlling the progression of breast cancer through the molecular crosstalk between skeletal muscle and cancer cells. In response to an exercise training bout, miRNAs are produced in the skeletal muscle and can translocate to the bloodstream inserted in extracellular vesicles, such as exosomes. c-miRNAs reach breast tumor through circulation and act as anti-cancer molecules, decreasing tumor proliferation, weight, and volume (Images from Freepik.com).

miRNA expression is modulated both acutely and chronically following exercise (D'Souza et al., 2017; Mooren et al., 2014; Nielsen et al., 2010; Telles et al., 2021; Uhlemann et al., 2014; Baggish et al., 2011; Banzet et al., 2013; Tonevitsky et al., 2013; Aoi et al., 2013; Sawada et al., 2013; Nielsen et al., 2014; Baggish et al., 2014; Mitchelson and Qin, 2015; Russell et al., 2013; Gomes et al., 2014). For example, miR-133 is canonically expressed in skeletal muscle (D'Souza et al., 2017; Nielsen et al., 2010; Telles et al., 2021; Mitchelson and Qin, 2015; Russell et al., 2013) and significantly increased in the bloodstream (i.e., c-miR-133) after an exercise bout (Banzet et al., 2013; Baggish et al., 2014; Gomes et al., 2014; Mooren et al., 2014; Nielsen et al., 2014). Interestingly, it was demonstrated that miR-133a expression is significantly reduced in five breast cancer cell lines (MCF-7, MDA-MB-231, BT-549, SK-BR-3, and T47D) compared to a normal breast cell line HBL-100, as well as in human breast cancer tissue compared to adjacent non-cancerous breast tissue (Cui et al., 2013). Thus, it has been suggested that this miRNA can translocate from skeletal muscle to circulation and to cancer cells, where it acts as an exercise-induced systemic factor downregulating tumor progression. Moreover, Pulliero, You (Pulliero et al., 2020) showed that an exercise bout modulates the expression of 14 c-miRNAs involved in pathways relevant to the control of cancer development and progression (e.g., oncogene and metastasis suppression, cell proliferation, apoptosis, cancer invasion) in healthy postmenopausal women (54–78 years old). Specifically, c-miR-206 and c-miR-30c expression were upregulated and downregulated, respectively. Interestingly, miR-206 transfection and anti-miR-30c silencing inhibit cell growth and increase apoptosis of MCF-7 breast cancer cells. It is noteworthy that miR-206 is also canonically expressed in the skeletal muscle (Mitchelson and Qin, 2015; Telles et al., 2021). Collectively, it is plausible to suggest that miRNAs can translocate from the muscle to the bloodstream in response to an exercise bout and regulate gene expression in pathways associated with tumor growth and suppression in breast cancer cells (Figure 1) (Dufresne et al., 2018). However, the aforementioned studies (Adams et al., 2018; Hagstrom and Denham, 2018; Alizadeh et al., 2019) only investigated the chronic c-miRNA response after an exercise training intervention in patients with breast cancer. Therefore, acute changes in c-miRNAs after an exercise bout (pre and post an exercise training intervention) remain to be elucidated in patients with breast cancer.

## Effect of Different Exercise Modes on c-miRNAs Expression in Breast Cancer

Exercise training can induce specific muscular adaptations depending on the exercise mode (Hawley, 2009; Hawley et al., 2014; Coffey and Hawley, 2016). For instance, resistance exercise training interventions maximize neuromuscular adaptations, such as muscle hypertrophy and strength (Campos et al., 2002; Mitchell et al., 2013; Bellamy et al., 2014; Nader et al., 2014; Damas et al., 2016; Morton et al., 2019). On the other hand, aerobic exercise training interventions improve aerobic muscle metabolism and cardiorespiratory fitness (e.g., aerobic power) (Maeda et al., 2001; Coffey and Hawley, 2007; Daussin et al., 2007; Sloth et al., 2013; Konopka et al., 2014; Milanovic et al., 2015). Besides different functional and morphological adaptations, distinct exercise modes can also affect myokines secretion (Trovato et al., 2019).

The expression of a number of miRNAs (e.g., miR-1, -21, -23a, -133a, -133b, -181a, -206, -378, and -486) is modulated in the human skeletal muscle (Camera et al., 2016; D'Souza et al., 2017; Davidsen et al., 2011; Drummond et al., 2008; Fyfe et al., 2016; Ogasawara et al., 2016; Telles et al., 2021; Russell et al., 2013; Rivas et al., 2014; Olsen et al., 2006) and bloodstream (Banzet et al., 2013; Baggish et al., 2014; Gomes et al., 2014; Nielsen et al., 2014) following distinct exercise intervention modes. Interestingly, some of these miRNAs may hamper tumor development, progression, and metastasis (Si et al., 2007; Zhu et al., 2008; Cui et al., 2013). In the breast cancer context, Hagstrom and Denham (Hagstrom and Denham, 2018) showed changes in the c-miR-133a-3p in high responders relative to low ones after a supervised resistance exercise training intervention (3 × 8–10 RM, three times per week for 16 weeks). On the other hand, the previously cited changes in c-miRNAs expression observed in the study of Alizadeh, Isanejad (Alizadeh et al., 2019) (i.e., lower expression of c-oncomiRs and greater increase in tumor suppressor miRNAs in the hormonal therapy-exercise training group) resulted from an aerobic exercise training intervention. Aerobic exercise was performed three times per week, for 12 weeks, using a high-intensity interval training protocol, composed of four sets of 4 min of uphill walking at an intensity of 90%–95% of the maximum heart rate, interspersed by 3 min of active recovery, at 50%–70% of the maximum heart rate. However, performing combined training (i.e., aerobic and resistance training performed in the same training period) has been deemed as the gold-standard non-pharmacological strategy to improve health and quality of life of women with breast cancer (Herrero et al., 2006; Schmitz et al., 2010; Mijwel et al., 2018; de Paulo et al., 2019). Also, combined training modulates skeletal muscle miRNA expression in healthy subjects (Camera et al., 2016; Fyfe et al., 2016; Telles et al., 2021). Moreover, the significant reductions in the viability of both MCF-7 and MDA-MB-231 breast cancer cell lines observed by Dethlefsen, Lillelund (Dethlefsen et al., 2016) were due to the incubation with serum from breast cancer survivors undergoing chemotherapy collected immediately after a 2-h acute combined exercise protocol. The exercise protocol consisted of 30 min of warm-up, 60 min of resistance training, and 30 min of a high-intensity interval exercise bout. Therefore, it is reasonable to suggest that combined exercise training can increase the expression of specific c-miRNAs related to the control of breast cancer cells. However, none of the cited studies (Adams et al., 2018; Hagstrom and Denham, 2018; Alizadeh et al., 2019) considered the potential of interventions combining aerobic and resistance exercise protocols in the same training program in breast cancer.

## Discussion

Exercise training has been deemed as a non-pharmaceutical strategy to counteract breast cancer (Campbell et al., 2019; Patel et al., 2019). The positive effect of exercise training is mainly driven by improvements in a set of outcomes (e.g., muscle hypertrophy and strength, cardiorespiratory fitness, body composition) ultimately associated with improved quality of life and reduced mortality risk in cancer patients (Herrero et al., 2006; Schmitz et al., 2010; Dethlefsen et al., 2017a; Adraskela et al., 2017; Kraschnewski and Schmitz, 2017; Hojman et al., 2018; Mijwel et al., 2018; Campbell et al., 2019; de Paulo et al., 2019; Patel et al., 2019). Emerging evidence suggests that exercise training can also directly affect breast tumor through changes in exercise-induced c-miRNA levels, which perform the molecular crosstalk between skeletal muscle and cancer cells (Hojman et al., 2011; Dethlefsen et al., 2016; Ruiz-Casado et al., 2017; Dufresne et al., 2018; Figueira et al., 2018; Hojman et al., 2018). However, existing studies did not have appropriate designs to determine the exercise effect *per se* on patients with breast cancer (Adams et al., 2018; Hagstrom and Denham, 2018; Alizadeh et al., 2019). Determining this effect is challenging as patients are usually undergoing cancer treatment (e.g., chemotherapy) or are survivors with no evidence of recurrent disease. Thus, isolating exercise training effects from other variables requires implementing an exercise intervention in the period between the cancer diagnostic and treatment commencement, named “window of opportunity” (Ligibel et al., 2019). This window allows investigating the exercise-induced changes in c-miRNAs expression, tumor biology (e.g., tumor metabolism, proliferation), and miRNAs expression in the tumor concomitantly.

Additionally, the c-miRNA responses of women with breast cancer or survivors have been investigated only chronically, pre and post exercise training interventions (Adams et al., 2018; Hagstrom and Denham, 2018; Alizadeh et al., 2019). As the direct effect of exercise training on controlling tumor development has been attributed to the repetitive and transient spikes in myokines release after each exercise bout (Dethlefsen et al., 2017a; Hojman et al., 2018), c-miRNA analysis should be performed before and immediately after it. Furthermore, as exercise-induced acute changes in muscle transcriptome may occur over time (Damas et al., 2018), it would be interesting to compare the changes in c-miRNA expression following an acute exercise bout performed before and after an exercise training intervention (i.e., baseline acute effect and post-training acute effect).

Finally, there is paucity of data regarding the changes in c-miRNAs expression induced by different exercise training modes. Importantly, c-miRNA expression is modulated following combined exercise protocols (Camera et al., 2016; Fyfe et al., 2016; Telles et al., 2021) and serum from breast cancer survivors, following a combined exercise bout, decreased cancer cells viability, in a cell culture model (Dethlefsen et al., 2016). Therefore, another perspective is to consider the use of combined resistance and aerobic exercise training protocol to investigate the acute/chronic c-miRNAs responses in patients with breast cancer.

In conclusion, evidence on the potential of exercise-induced c-miRNAs in breast cancer control is growing, but the topic is far from being elucidated. Here, we summarized the current knowledge into the topic and suggested perspectives for future experimental designs to investigate the direct role of exercise-induced c-miRNAs in breast cancer. Advances in knowledge towards the mechanisms underpinning the exercise-induced c-miRNAs in breast cancer can be important to determine new biomarkers related to mitigating the burden of the disease. In addition, the discovery of molecules related to breast tumor control can help the development of new technologies with the potential to treat patients with breast cancer.

## Data Availability

The original contributions presented in the study are included in the article/Supplementary Material, further inquiries can be directed to the corresponding author.

## Abbreviations

BALB/c, Concatenation of Bagg and Albino; c-miRNAs, Circulatory Micro Ribonucleic Acid; ERα, Estrogen receptor alpha; HIF-1, Hypoxia-inducible factor 1-alpha; miRNA, Micro Ribonucleic Acid; mRNA, Messenger Ribonucleic Acid; oncomiR, Oncogenic Micro Ribonucleic Acid; RM, Repetition Maximum; RNAs, Ribonucleic Acid.
